# Identification of prognostic and metastasis-related alternative splicing signatures in hepatocellular carcinoma

**DOI:** 10.1042/BSR20201001

**Published:** 2020-07-15

**Authors:** Runzhi Huang, Gaili Yan, Hanlin Sun, Jie Zhang, Dianwen Song, Rui Kong, Penghui Yan, Peng Hu, Aiqing Xie, Siqiao Wang, Juanwei Zhuang, Huabin Yin, Tong Meng, Zongqiang Huang

**Affiliations:** 1 Department of Orthopedics, The First Affiliated Hospital of Zhengzhou University, 1 East Jianshe Road, Zhengzhou, China; 2 Division of Spine, Department of Orthopedics, Tongji Hospital Affiliated to Tongji University School of Medicine, 389 Xincun Road, Shanghai, China; 3 Tongji University School of Medicine, Tongji University, 1239 Siping Road, Shanghai 200092, China; 4 Department of Orthopedics, Shanghai General Hospital, School of Medicine, Shanghai Jiaotong University, 100 Haining Road, Shanghai, China; 5 Department of Gastroenterology, Shanghai Tenth People’s Hospital, Tongji University School of Medicine, Shanghai 200072, China; 6 School of Ocean and Earth Science, Tongji University, 1239 Siping Road, Shanghai 200092, China

**Keywords:** alternative splicing, hepatocellular carcinoma, metastasis, prognosis

## Abstract

As the most common neoplasm in digestive system, hepatocellular carcinoma (HCC) is one of the most important leading cause of cancer deaths worldwide. Its high-frequency metastasis and relapse rate lead to the poor survival of HCC patients. However, the mechanism of HCC metastasis is still unclear. Alternative splicing events (ASEs) have a great effect in cancer development, progression and metastasis. We downloaded RNA sequencing and seven types of ASEs data of HCC samples, in order to explore the mechanism of ASEs underlying tumorigenesis and metastasis of HCC. The data were taken from the The Cancer Genome Atlas (TCGA) and TCGASpliceSeq databases. Univariate Cox regression analysis was used to determine a total of 3197 overall survival-related ASEs (OS-SEs). And based on five OS-SEs screened by Lasso regression, we constructed a prediction model with the Area Under Curve of 0.765. With a good reliability of the model, the risk score was also proved to be an independent predictor. Among identified 390 candidate SFs, Y-box protein 3 (YBX3) was significantly correlated with OS and metastasis. Among 177 ASEs, ATP-binding cassette subfamily A member 6 (ABCA6)-43162-AT and PLIN5-46808-AT were identified both associated with OS, bone metastasis and co-expressed with SFs. Then we identified primary bile acid biosynthesis as survival-related (KEGG) pathway by Gene Set Variation Analysis (GSVA) and univariate regression analysis, which was correlated with ABCA6-43162-AT and PLIN5-46808-AT. Finally, we proposed that ABCA6-43162-AT and PLIN5-46808-AT may contribute to HCC poor prognosis and metastasis under the regulation of aberrant YBX3 through the pathway of primary bile acid biosynthesis.

## Introduction

Hepatocellular carcinoma (HCC) is the most common primary liver tumor and one of the most important lethal malignancies worldwide [1]. In China, the incidence of HCC has been increasing continually and the 5-year survival rate has been less than 15% in the past decade [2–4]. Currently, optional treatments for liver cancer are very limited and the prognosis is generally unsatisfactory [5,6]. The highly metastatic capability and recurrence rate of HCC result in the low survival rate of HCC patients. Therefore, HCC has become a severe public health problem worldwide [7]. Although many advances have been achieved in the diagnosis and treatment of HCC, the overall prognosis for HCC patients remains poor. Tumor metastasis contributes greatly to the poor prognosis of HCC patients. Current therapeutic strategies for HCC have been demonstrated to promote HCC metastasis instead of repressing it [8]. Therefore, investigations of the molecular mechanisms underlying HCC metastasis are urgently needed to develop potential therapeutic strategies to target HCC metastasis [9].

Alternative splicing (AS) is an important post-transcriptional regulatory mechanism that increases protein diversity [10]. AS of pre-mRNA transcribed from a single gene can generate isoforms with distinct structures and functions [11]. Approximately 95% of the genes in the human genome undergo AS [12]. Aberrant AS can play a role in cancer development and resistance to therapy [13–15]. AS events (ASEs) could therefore function as diagnostic or prognostic biomarkers in various cancers. Additionally, cancer-specific splice isoforms or splicing factors could be therapeutic targets. In the past few years, emerging data have suggested that the cancer progression and metastasis are specifically associated with a plethora of mRNA isoforms [16].

Although multiple studies have revealed the critical role of many genes in the development and progression of HCC, most previous studies focused on alteration in transcriptome level. Analysis of post-transcriptional process is largely ignored, especially the potential roles of ASEs in the pathogenesis of HCC.

In our study, a comprehensive analysis was performed to characterize AS profiling and we constructed a prognostic model based on metastasis-related and overall survival-related ASEs (OS-SEs) for HCC. Additionally, Pearson correlation analysis was used to identify metastasis-associated ASEs as well as the corresponding SFs and pathways to elucidate the latent metastasis mechanism in HCC. Our results proved that the hypothetical constructed model has an important significance in prognostic prediction of HCC, which might assist oncologists in clinical management. Furthermore, a new sneak molecular mechanism and three potential therapeutic targets for HCC metastasis (YBX3 (Y-box protein 3), ABCA6, PLIN5) were both identified among the present study. In the present study, the active search about AS profiling has identified three potential therapeutic targets for HCC metastasis (YBX3, ABCA6, PLIN5). Meanwhile, the underlying molecular mechanism was also investigated.

## Methods

### Data extraction

RNA-seq data and SFs of 390 HCC cases were downloaded from the The Cancer Genome Atlas (TCGA) Data Portal (https://tcgadata.nci.nih.gov/tcga/). Meanwhile, we retrieved seven types of ASE data (spare receptor sites, AA; exon skip, ES; alternative terminator, AT; mutually exclusive exons, ME; reserved introns, RI; Alternative donor site, AD; alternative promoter, AP) from the TCGASpliceSeq database (https://bioinformatics.mdanderson.org/TCGASpliceSeq/). Samples with more than 25 percent of missing percent splicing (PSI) values were excluded.

### Identification of OS-SEs

For ASEs, samples without follow-up records and samples with mean and standard deviation of PSI less than 0.05 and 0.01 were excluded, and K-nearest neighbor algorithm was used to correct for ASEs lacking expression data. Afterward, the univariate Cox regression analysis of integrated ASE and clinical data wereused to assess the prognostic value of each screened ASE. To demonstrate the prognosis-related and -unrelated ASEs holistically, the volcano plot was developed. Meanwhile, UpSet plot showed the OS-SEs. To display the top 20 OS-SEs of AA, AD, AP, AT, ES, ME and RI, seven bubble plots were generated, where the color and size of bubbles represent the overall survival value of ASEs.

### Construction of the prognostic model based on the OS-SEs

First, to enhance the adaptability of the prognostic model constructed below, Lasso regression was performed to filter the top 20 significant prognostic OS-SEs. The significance of each OS-SEs screened by Lasso regression can be evaluated by using a multivariate Cox regression model with β-value, which indicated the regression coefficient of every integrated OS-SE in the model. Risk score was thus acquired by the following formula:

$$\sum_{i=1}^{n}\beta \text{i}\times \text{PSI}$$

The samples were scored by two risk groups based on the median risk score and were reordered according to the risk score, which enabled the risk curve, scatterplot and expression heatmap be generated. Then we assessed the accuracy of the model by using the area under the receiver operator characteristic (ROC) curve. In addition, the identification of differences between high-risk and low-risk groups can also be resolved by Kaplan–Meier survival analysis.

To assess whether the risk score, as well as age, gender, grade, stage and TNM stage was independent prognostic factor, univariate and multivariate Cox regression analyses can be applied by modifying the baseline information.

### Construction of the interaction and correlation network

In the SpliceAid2 database, 377 splicing factors were retrieved [17]. Pearson correlation analysis was utilized to inquiry the interaction and correlation between SFs and OS-SEs. The regulation network of SFs and OS-SEs was constructed by Cytoscape (3.7.1) with the exclusion criteria of the regulation pairs with *P*>0.001 and the absolute value of correlation coefficient < 0.75 [18]. In the network, we define the positive and negative regulations as red and green lines, respectively, the low and high risk of OS-SEs as purple and red, and OS-SEs and SF as ovals and arrows.

### Identification of metastasis- and/or stage-related OS-SEs

To identify the OS-SEs significantly associated with TNM stage, metastasis or both of them, Kruskal–Wallis test and Mann–Whitney–Wilcoxon test were performed, and results were displayed by beeswarm plots. Moreover, the regulation network also contains these metastasis-/stage-related OS-SEs.

### Co-expression analysis between ASEs and signaling pathways

To screen for prognosis-associated signal pathways identified by Gene Set Variation Analysis (GSVA), univariate Cox analysis was performed [19]. Then, to determine the possible downstream mechanisms of OS-SEs, we included key OS-SEs (distant metastasis/bone metastasis/stage-related) and the Kyoto Gene and Genomic Encyclopedia (KEGG) pathway (prognosis-related) into co-expression analysis.

### Online database validation

To minimize bias, multiple databases were used to detect gene and protein expression levels of key biomarkers at the tissue levels, including the UALCAN [20], Kaplan–Meier plotter [21], LinkedOmics [22], SurvExpress [23], Firebrowse [24], GEPIA, CCLE, Oncomine, String.

### Statistical analysis

All statistical analyses were applied by R version 3.5.1 (Institute for Statistics and Mathematics, Vienna, Austria; www.r-project.org) (Package: impute, UpSetR, ggplot2, rms, glmnet, preprocessCore, forestplot, survminer, survivalROC, beeswarm). Two-tailed *P*<0.05 was regarded statistically significant.

## Results

### Analysis of ASEs data and identification of OS-SEs in HCC

**Figure 1:**
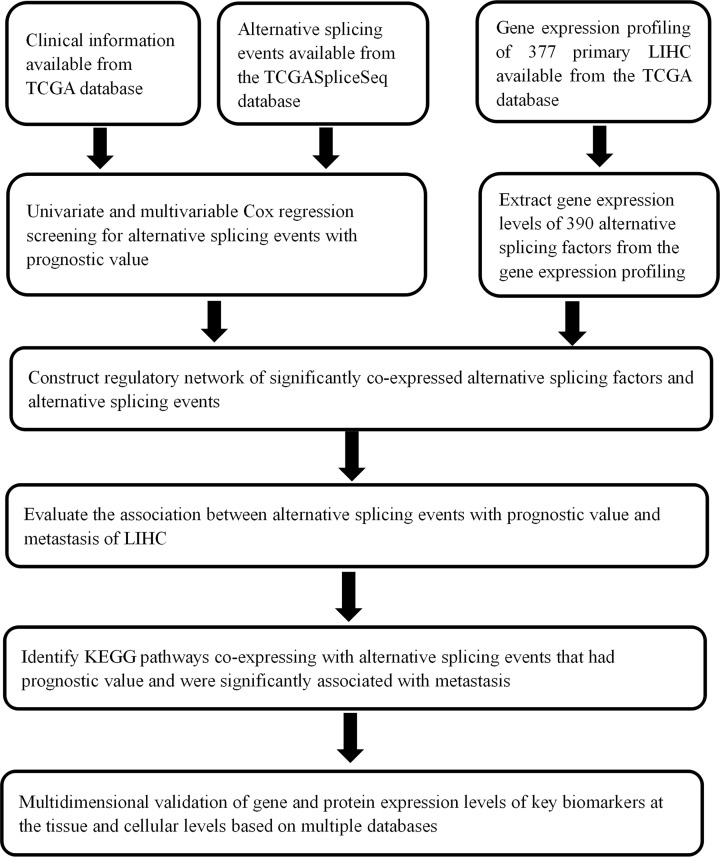
Flowchart of the study

**Figure 2:**
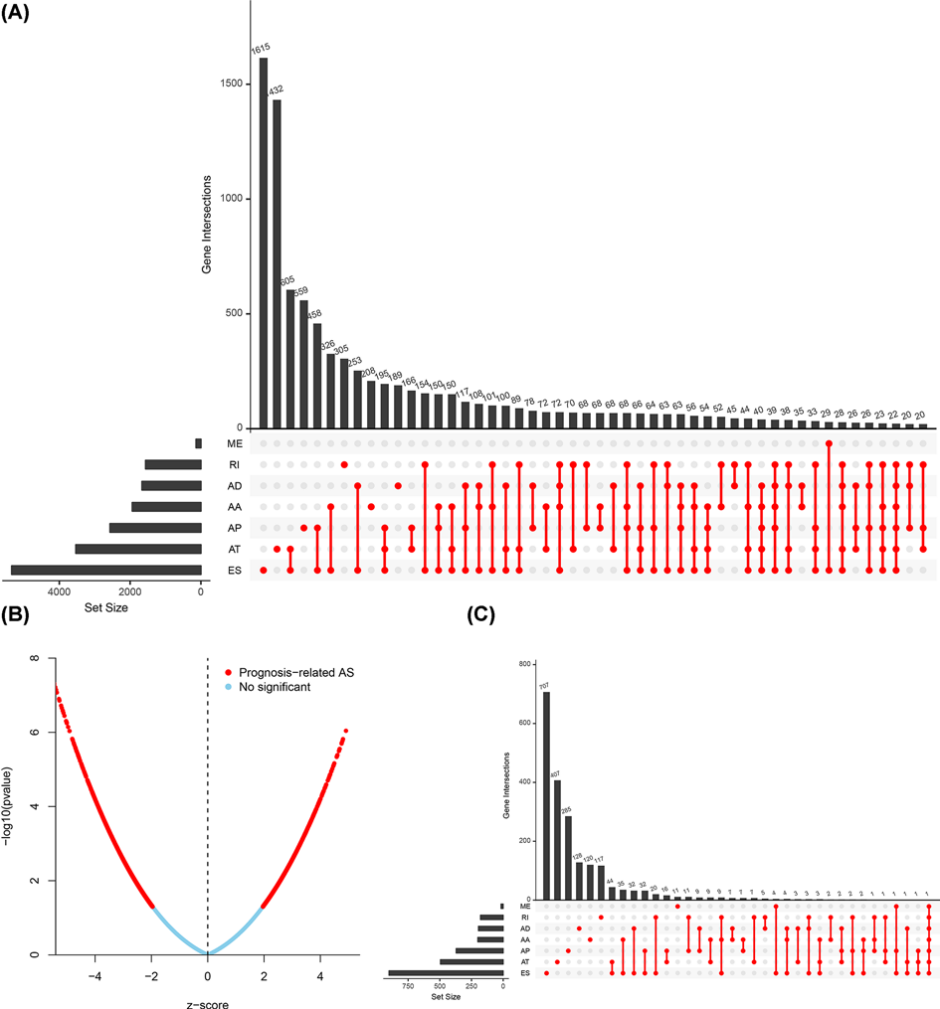
The Upset plots and volcano plot of HCC ASEs A total of 34163 ASEs in seven AS patterns (**A**). The volcano plot exhibited the result of univariate Cox regression analysis (**B**). A total of 3197 OS-SEs identified by univariate Cox regression analysis in seven AS patterns (**C**).

**Figure 3:**
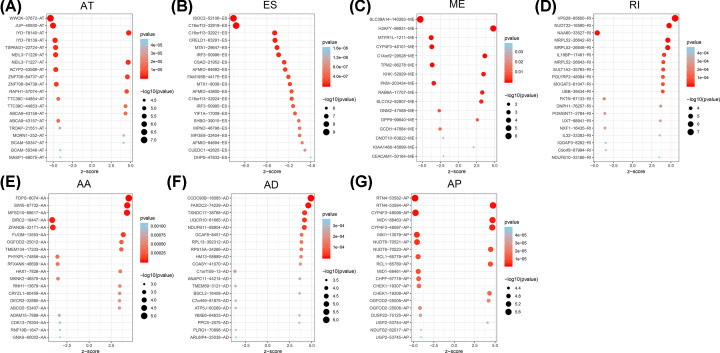
The bubble plots of HCC ASEs The bubble plots displayed the top 20 OS-SEs of each type (**A**–**G**).

The flow chart of the present study is illustrated in Figure 1. ASEs data of 377 HCC samples were collected from TCGASpliceSeq (https://bioinformatics.mdanderson.org/TCGASpliceSeq/) and we identified a total of 34163 ASEs in 8985 genes among the HCC cases: 12327 ES events in 5343 genes, 8087 AT events in 3532 genes, 6352 AP events in 2566 genes, 2666 AA events in 1937 genes, 2331 AD events in 1663 genes, 2263 RI events in 1561 genes and 137 ME events in 135 genes. Therefore, multiple AS types could occur in the same genes (Figure 2A). We used univariate Cox regression analysis for determining OS-ASEs by FDR < 0.05 and 3197 events were found (Figure 2B). Additionally, based on 3197 events, UpSet plots were constructed to visualize the relationship of seven types ASEs and genes (Figure 2C) and bubble plots were generated to show the top 20 OS-SEs in seven types, respectively (Figure 3A–G).

### Prognostic predictors of OS in HCC

**Figure 4:**
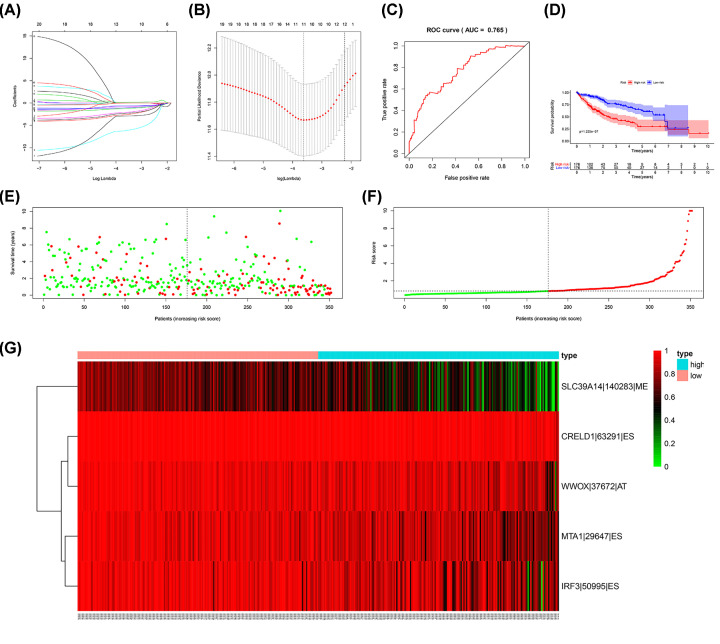
Construction and assessment of the prognosis model The Lasso regression for the 20 most significant OS-ASEs. (**A,B**) We constructed a predict model based on eight OS-SEs screened by Lasso regression, the ROC curves to evaluate the accuracy of the model and the area under the curves is 0.765. (**C**) K–M survival analysis for demonstrating risk score able to predict prognosis in patients with HCC (**D**). The risk curve for ranking patients by value of risk score (**E**). The green and red dots of the scatter plot representing survival and death, respectively (**F**). The heat map for PSI of five OS-ASEs screened by Lasso regression (**G**).

**Figure 5:**
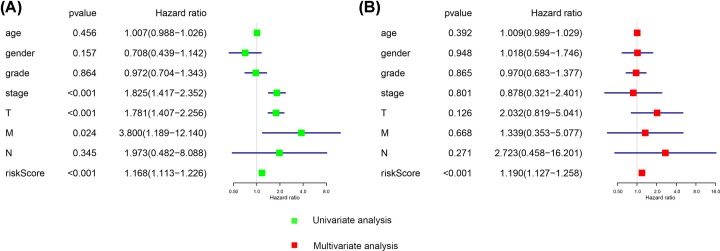
Estimation of independent prognostic value Univariate (**A**) and multivariate (**B**) Cox regression analyses suggested risk score can be used as independent prognostic factor for HCC.

After filtration of the Lasso regression for the 20 most significant OS-SEs, ISOC2/52106/ES, CRELD1/63291/ES, MTA1/29647/ES, WWOX/37672/AT, SLC39A14/140283/ME, MTX1/8038/ES, AFMID/43800/ES, IRF3/50995/ES were integrated to construct a prediction model (Figure 4A,B). Next, we generated ROC curves to evaluate the accuracy of the prediction model and the area under the curves is 0.765 (CI = 95%) (Figure 4C). Based on the two subgroups divided by risk score, Kaplan–Meier survival analysis displayed that there is a poor prognosis for high-risk group which indicated that the prediction model had a good value (*P*= .225E-7) (Figure 4D). The risk curve showed the result of ranking patients by value of risk score (Figure 4E). The green and red dots of the scatter plot represent survival and death, respectively, and the result disclosed there was a higher mortality in a high-risk group (Figure 4F). Besides, we generated heat map to display the expression level for five OS-SEs (SLC39A14-14028-ME, CRELD1-63291-ES, WWO-37672-AT, MTA1-29647-ES and IRF3) filed by Lasso regression, and all of them were higher in the high-risk group (Figure 4G). Furthermore, we used univariate as well as multivariate Cox regression analyses to estimate the independent prognostic value of age, gender, grade, stage and risk score. Both results suggested riskScore could be considered as an independent prognostic factor (univariate model: HR = 1.168, 95% CI (1.113–1.226), *P*<0.001; multivariate model: HR = 1.190, 95% CI (1.127–1.258), *P*<0.001) (Figure 5).

### Construction of the regulatory network between ASEs and SFs and identification of metastasis-related ASEs

**Figure 6:**
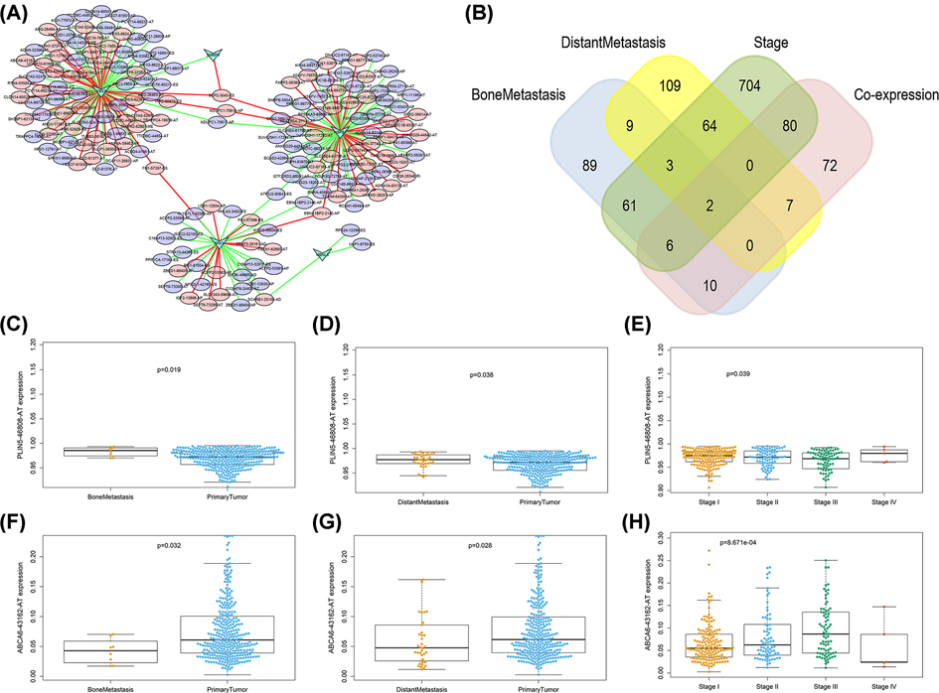
Regulatory network between OS-SEs and co-expressed SFs The results indicated five SFs (HSPB1, MBNLS, MSI, SNRPN, YBX3) were correlated with 177 ASEs. Of the 187 pairs of corelation cases, 104 pairs were negatively regulated (green lines) and 83 pairs were positively regulated (red lines). Of the 177 ASEs, 79 were high-risk events (red ovals) and 98 were low-risk events (green ovals) (**A**). Venn plot for displaying OS-SEs associated metastasis and clinical status, among the 177 ASEs, 18 ASEs were significantly connected with bone metastasis, ABCA6-43162-AT and PLIN5-46808-AT were identified to be both associated with OS (*P*=0.041, *P*=0.048), bone metastasis (*P*=0.032, *P*=0.019) and co-expressed with SFs (cor = 0.410, *P*=1.05E-15; cor = −0.416, *P*=3.83E-16) (**B**). Beeswarm plots demonstrating OS-SEs, ABCA6-43162-AT and PLIN5-46808-AT, were significantly associated with metastasis and clinical status (**C–H**).

Since AS is the outcome of SFs, in order to find SFs-associated ASEs, Pearson correlation analysis between OS-SEs and 390 SFs was performed. The Cytoscape was then implemented to generate regulation network. The results indicated that five SFs (HSPB1, MBNLS, MSI, SNRPN, YBX3) were correlated with 177 ASEs. Among 187 pairs of correlation cases, 104 pairs were negatively regulated (green lines) and 83 pairs were positively regulated (red lines). Of the 177 ASEs, 79 were high-risk events (red ovals) and 98 were low-risk events (green ovals) (Figure 6A). Among the 177 ASEs, 18 ASEs were significantly connected with bone metastasis, especially, ABCA6-43162-AT and PLIN5-46808-AT, which were both associated with OS (*P*=0.041, *P*=0.048), bone metastasis (*P*=0.032, *P*=0.019) and co-expressed with YBX3 (correlation coefficient = 0.410, *P*<0.001; correlation coefficient = −0.416, *P*<0.001) (Figure 6B–H).

### Integrated analysis of survival-related KEGG pathways and metastasis-related ASEs

**Figure 7:**
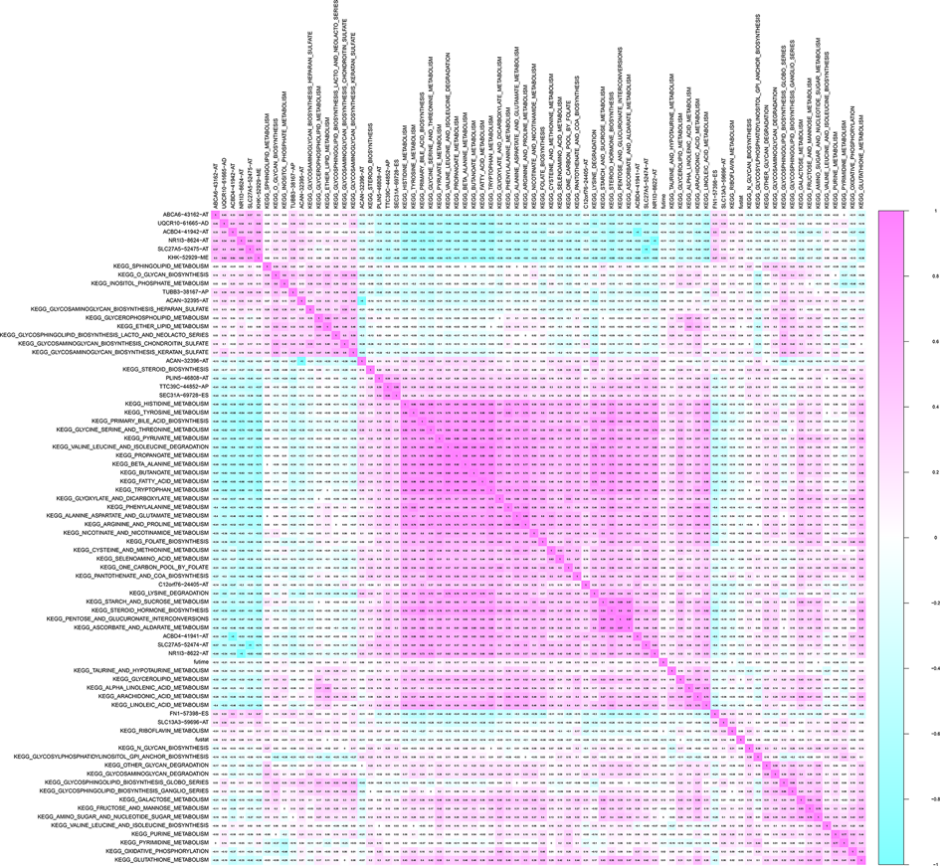
Co-expression heat map between KEGG pathway identified by GSVA and OS-SEs related with metastasis ABCA6-43162-AT and PLIN5-46808-AT were both correlated with primary bile acid biosynthesis (R = −0.49; R = 0.36).

Through GSVA, we identified survival-related KEGG pathways with univariate regression analysis. With the co-expression analysis, some significant correlation patterns between survival-related KEGG pathways and ASEs intersecting of bone metastasis and regulatory network were shown in Figure 7, and ABCA6-43162-AT and PLIN5-46808-AT were both correlated with primary bile acid biosynthesis (R = −0.49; R = 0.36).

### External validation

**Figure 8:**
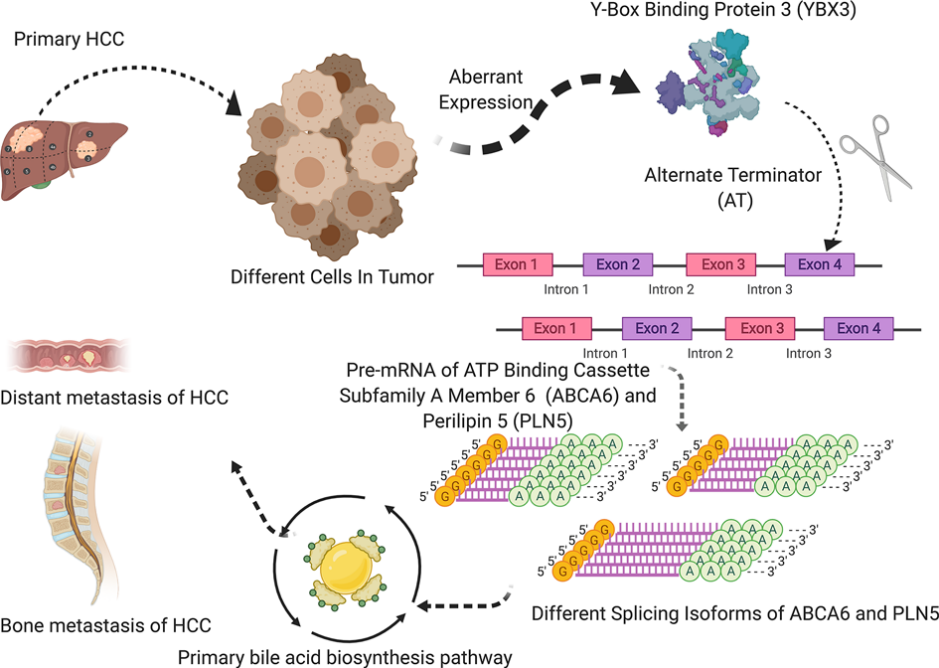
The speculative mechanism diagram of the article The speculative mechanism diagram included YBX3, ABCA6-43162-AT, PLIN5-46808-AT and primary bile acid biosynthesis pathway.

The top five genes for pathway of primary bile acid biosynthesis were CH25H, AMACR, AKR1D1, CYP27A1, CYP46A1 which were identified by Pathcard database (http://pathcards.genecards.org/). Then to enhance the validity of our results, we performed a multidimensional validation for verifying prognostic value of ABCA6, PLIN5, YBX3 and the five genes mentioned above. All the results are shown in Table 1. ABCA6, PLIN5, AMACR, AKR1D1 CYP27A1 and CYP46A1 are differently expressed genes among clinical stages in UALCAN (Supplementary Figure S1). In the Human Protein Atlas, the expression of YBX3, AKR1D1 and CYP27A1 are lower in HCC than normal liver but the AMACR are opposite (Supplementary Figure S2). Most of the genes are significantly associated with survival in Kaplan–Meier (Supplementary Figure S3). ABCA6, PLIN5, AKR1D1 and CYP27A1 were significantly related to survival and clinical stages in Linkedomics (Supplementary Figure S4). At the tissue level, YBX3, ABCA6, PLIN5, CH25H, AMACR, AKR1D1, CYP27A1 and CYP46A1 were all significantly related to survival in SurvExpress (Supplementary Figure S5). The differential expression of ABCA6, PLIN5, AKR1D1, CYP27A1 are related with survival and clinical stages in GEPIA (Supplementary Figure S6). At the cellular level, YBX3 and CH25H were highly expressed in liver cancer cell lines, and ABCA6, PLIN5, AMACR, AKR1D1, CYP27A1, CYP46A1 were highly expressed in liver cancer cell lines in CCLE (Supplementary Figure S7). PLIN5, AMACR and YBX3 expressed differently in multiple studies and multiple cancers in Oncomine (Supplementary Figure S8). Finally, Figure 8 summarizes the speculative mechanism diagram including YBX3, ABCA6-43162-AT, PLIN5-46808-AT and primary bile acid biosynthesis pathway.

**Table 1:** The external validation of YBX3, ABCA6, PLIN5, CH25H, AMACR, AKR1D1, CYP27A1, CYP46A1

Database	YBX3 (splicing factor gene)	ABCA6 (ASEs gene)	PLIN5 (ASEs gene)	CH25H (pathway gene)	AMACR (pathway gene)	AKR1D1 (pathway gene)	CYP27A1 (pathway gene)	CYP46A1 (pathway gene)
**UALCAN**	Expression: *P*<0.001 Stage: *P*=0.001	Expression: *P*=0.002 Stage: *P*<0.001	Expression: *P*<0.001 Stage: *P*<0.001	Expression: *P*=0.67 Stage: *P*=0.24	Expression: *P*<0.001 Stage: *P*<0.001	Expression: *P*<0.001 Stage: *P*<0.001	Expression: *P*<0.001 Stage: *P*<0.001	Expression: *P*=0.23 Stage: *P*=0.018
**The Human Protein Atlas**	Tumor; media Normal; not detected	None	None	None	Tumor; media Normal; high	Tumor; media Normal; not detected	Tumor; media Normal; not detected	Tumor; media Normal; not detected
**Kaplan–Meier plotter**								
**Best cutoff**	*P*=0.17	*P*<0.001	*P*<0.001	*P*=0.016	*P*=0.100	*P*<0.001	*P*<0.001	*P*=0.225
**Median value**	*P*=0.51	*P*=0.118	*P*=0.073*P*=	*P*=0.316	*P*=0.409	*P*<0.001	*P*<0.001	*P*=0.688
**LinkedOmics**	K–M analysis: *P*=0.463 Stage: *P*=0.607	K–M analysis: *P*=0.003 Stage: *P*=0.041	K–M analysis: *P*<0.001 Stage: *P*=0.002	K–M analysis: *P*=0.066 Stage: *P*=0.370	K–M analysis: *P*=0.082 Stage: *P*=0.924	K–M analysis: *P*=0.004 Stage: *P*=0.001	K–M analysis: *P*<0.001 Stage: *P*<0.001	K–M analysis: *P*=0.614 Stage: *P*=0.421
**SurvExpress**	K–M analysis: *P*=0.039	K–M analysis: *P*=0.036	K–M analysis: *P*=0.007	K–M analysis: *P*=0.025	K–M analysis: *P*=0.038	K–M analysis: *P*=0.019	K–M analysis: *P*=0.015	K–M analysis: *P*=0.011
**GEPIA**	Tumor low Nomal low KM: *P*=0.45 Stage: *P*=0.171	Tumor media Nomal high KM: *P*=0.041 Stage: *P*=0.002	Tumor low Nomal high KM: *P*=0.006 Stage: *P*=0.038	Tumor low Nomal low KM: *P*=0.69 Stage: *P*=0.945	Tumor low Nomal low KM: *P*=0.36 Stage: *P*=0.72	Tumor low Nomal high KM: *P*=0.05 Stage: *P*=0.002	Tumor high Nomal high KM: *P*=0.009 Stage: *P*<0.001	Tumor low Nomal high KM: *P*=0.94 Stage: *P*=0.585
**CCLE**	Tumor high	Tumor low	Tumor low	Tumor low	Tumor high	Tumor low	Tumor low	Tumor low
**Oncomine**								

Abbreviations: ABCA6, ATP-binding cassette subfamily A member 6; AKR1D1, aldo-keto reductase family 1 member D1; AMACR, α-methylacyl-CoA racemase; CH25H, cholesterol 25-hydroxylase; CYP27A1, cytochrome P450 family 27 subfamily A member 1; CYP46A1, cytochrome P450 family 46 subfamily A member 1; PLIN5, Perilipin 5; YBX3, Y-box binding protein 3.

## Discussion

HCC was the commonest primary liver tumor and related to unsatisfactory prognosis [1]. Previous studies identified some key genes in HCC development and progression, however, fewer focused on analysis of post-transcription process, especially HCC metastasis [7,8]. Under the regulation of AS, a single gene could generate isoforms with distinct structures and functions [11,12]. Furthermore, aberrant AS could contribute to the production of onco-protein, leading to tumorigenesis, progression and metastasis of HCC [10–13].

In the present study, we totally identified 3197 HCC OS-SEs by univariate Cox regression analysis and then we constructed a prognosis model based on the eight OS-SEs filtered by the Lasso regression. Besides, risk score was demonstrated to be used as an independent prognostic factor. More importantly, among the OS-SEs, we identified two bone metastasis-related ASEs (ABCA6-43162-AT and PLIN5-46808-AT) co-expressing with SF YBX3 and pathway of primary bile acid biosynthesis. Based on the results, we supposed YBX3 regulated ABCA6-43162-AT and PLIN5-46808-AT of the primary bile acid biosynthesis pathway resulting in HCC metastasis.

YBX3upregto the Y-box binding protein family. The Y-box binding proteins play diverse regulatory functions, including regulation of transpription and translation, and are considered to be contributing to cell proliferation and transformation [25]. These proteins contain a highly conserved nucleic acid-binding motif called the cold shock domain (CSD). In YB proteins, the CSD is flanked by the N-terminal alanine/proline-rich(A/P) domain and the extended C-terminal domain (CTD) containing positively and negatively charged clusters of amino acids [26]. In all three members of the family, CSDs show more than 90% identity, while CTDs are close in amino acid composition and distribution of charged clusters. CTDs of YB protein family members from different vertebrates show high homology (approximately 60% identity) [27]. The CSD enables Y-box proteins to interact with both DNA and RNA to control the transcription and translation of specific genes [28,29]. Y-Box binding proteins can fall into three subfamilies, YBX1, YBX2 and YBX3.

YB-1, as the prototype member of this family, its nuclear expression has been reported to be correlated with advanced stages of malignant diseases, such as breast cancer [30], non-small lung cancer [31], thyroid cancer [32] and colorectal cancer [33]. Notably, reports have linked YB-1 with cancer cell invasiveness, promoting metastasis of liver cancer [34]. The studies of this protein have largely contributed to the recognition of the wide functional abilities of YB proteins. Since the composition, structure and order of YB-1 and YB-3 were similar to each other, YBX3 might similarly play a role in transport pathways between nucleus and cytoplasm and replace YB-1 in the regulation of mRNA translation and stability. In our study, YBX3 was the only aberrant SF co-expressing with ABCA6-43162-AT and PLIN5-46808-AT which are high risk OS-ASEs for HCC. Epidemiologically, chronic infection with hepatitis B virus or hepatitis C virus is closely related with the development of HCC. According to one study, dbpA could accelerate the process of inflammation-induced hepatocarcinogenesis [35]. And it has been reported that the T-to-G transversion in the dbpA promoter region was suggested to be a predisposing factor for the progression of HCC [36]. A similar view was also reported in another paper: enhanced expression of dbpA gene might be one of the accelerating factors in the hypercarcinogenic state, by provoking the genetic instability which is a prerequisite event leading to hepatocarcinogenesis [37].

ATP-binding cassette (ABC) transporters have been acknowledged as important players in the processes of transmembrane transport which utilize ATP hydrolysis energy to transport various substrates including lipid, amino acids, peptides, ions and sugars [38–40]. As an intracellular member of the ABC A subfamily, ABCA6 mediated the translocation of various physiological lipid compounds. Besides, ABCA6 was the first gene of ABC A subfamily to be described in completion because of its unique genomic organization and high sequence homology. A study indicated that ABCA6 may play a part in lipid transport [41]. Additionally, Kaminski et al. found mutation of the ABC A proteins were related to a variety of diseases and Elisabeth et al. found ABCA6 variant associated with cholesterol levels [42,43], which is known to be the inhibitor of HCC invasion and metastasis [44].

Similarly associated with lipid, PLIN5 belongs to the family of perilipins consisting of five distinct members (PLIN1-5), which are major structural proteins located on the surface of lipid droplets known to serve as factors for lipid homeostasis by modulating lipid storage, with crucial roles in diseases characterized with lipid manifestations [45,46]. In our study, PLIN5-46808-AT was more common in primary sites of HCC with metastasis than primary HCC. One study indicate that Lipocalin 2 (LCN2) is a key modulator of hepatic lipid homeostasis that controls the formation of intracellular lipid droplets by regulating PLIN5 expression [47]. The formation of intracellular lipid droplets, which PLIN5 involved, may lead to progression of non‐alcoholic fatty liver disease (NAFLD), the second leading etiology of HCC and currently the most common cause of chronic liver disease [48,49]. Furthermore, LCN2 and PLIN5 has been found highly expressed specifically in tumoral areas of HCC livers in human patients as well as in experimental HCC mouse models [50]. Importantly, mounting evidence proved that abnormal lipid metabolism has a negative role in HCC and other cancer [51–53]. Thus, we conjecture that ABCA6 and PLIN5 promote HCC progression by some lipid biosynthesis process.

The co-expression between the ASEs of ABCA6-43162-AT, PLIN5-46808-AT and the pathway of bile acid biosynthesis in the present study has also proved both the ASEs were related to HCC metastasis by affecting lipid metabolism. Bile acids acted as an emulsifier and helped to absorb and transport lipids. They were regarded as key signal molecules involving in lipid metabolism. Our study found that the pathway of primary bile acid biosynthesis was co-expressed with HCC metastasis-related ASEs (ABCA6-43162-AT and PLIN5-46808-AT) and we inferred that the pathway was involved in metastasis of HCC. A multicenter international study showed that primary biliary cirrhosis significantly promoted HCC initiation and this effect might be related to the activation of inflammatory signaling [54,55].

Our data revealed that ABCA6, PLIN5 and the pathway were all associated with lipid in various aspects. The further result of String database also demonstrated that there are extensive connections among our hypothesis members. ABCC3, establishing broad links between ABCA6 and the pathway genes, may play a part on transportation of organic anions in biliary and intestinal excretion. Besides, it is associated with Extrahepatic Cholestasis [56]. PPARA, link relay station of PLIN5 and pathway gene, acts as a transcription factor regulates fatty acid metabolism genes, such as intracellular binding and fatty acid transport [57].

There are incontrovertibly some limitations in our study. First of all, the data we collected from public databases are limited so that clinical information is not comprehensive and may cause potential bias and errors. Second, the present study is a multidimensional correlation study rather than a specific biological mechanism. Last but not least, single bioinformatics analysis lacks high credibility. Therefore, cell and animal experiments and clinical trials still need to be done to verify the findings. Although there are limitations, for all we know, our study was the first to link ASEs effects on lipid-to-lipid effects on HCC metastasis. In the future, we will concentrate on the splicing isoforms of ABCA6-43162-AT and PLIN5-46808-AT to further explore mechanism of HCC metastasis.

In conclusion, risk score calculated by the predict model can be used as an independent prognostic factor for HCC. We proposed that ABCA6-43162-AT and PLIN5-46808-AT are regulated by YBX3 via the pathway of primary bile acid biosynthesis, and this regulatory relationship can lead to a poor prognosis and clinical outcome in the progression of HCC.

## Supplementary Material

Supplementary Figures S1-S8 and Supplementary Table S1

## Data Availability

The datasets generated and/or analyzed during the current study are available in the Supplementary Material and TCGA-LGG program (https://portal.gdc.cancer.gov).

## Abbreviations

ABC: ATP-binding cassette
ABCA: ATP-binding cassette subfamily A member
AD: alternative donor
AP: alternative promoter
AS: alternative splicing
ASE: AS event
AT: alternative terminator
CI: confidence interval
CSD: cold shock domain
CTD: C-terminal domain
dbpA: DNA binding protein A
FDR: false discovery rate
GSVA: Gene Set Variation Analysis
HCC: hepatocellular carcinoma
HR: hazard ratio
KEGG: Kyoto Gene and Genomic Encyclopedia
LCN2: Lipocalin 2
ME: mutually exclusive exon
OS-SE: overall survival-related ASE
Pre-mRNA: precursor mRNA
RI: reserved intron
ROC: receiver operator characteristic
TCGA: The Cancer Genome Atlas
TNM: tumor, node, metastasis
YBX3: Y-box protein 3
