# Supplementary material for: Identification of prognostic and metastasis-related alternative splicing signatures in hepatocellular carcinoma
Source: Biosci Rep. 2020 Jul 15;40(7):BSR20201001. doi: 10.1042/BSR20201001 (PMC7364508; doi:10.1042/BSR20201001)
Supplement: Supplementary Figures S1-S8 and Supplementary Table S1 [file BSR-2020-1001_supp.pdf]

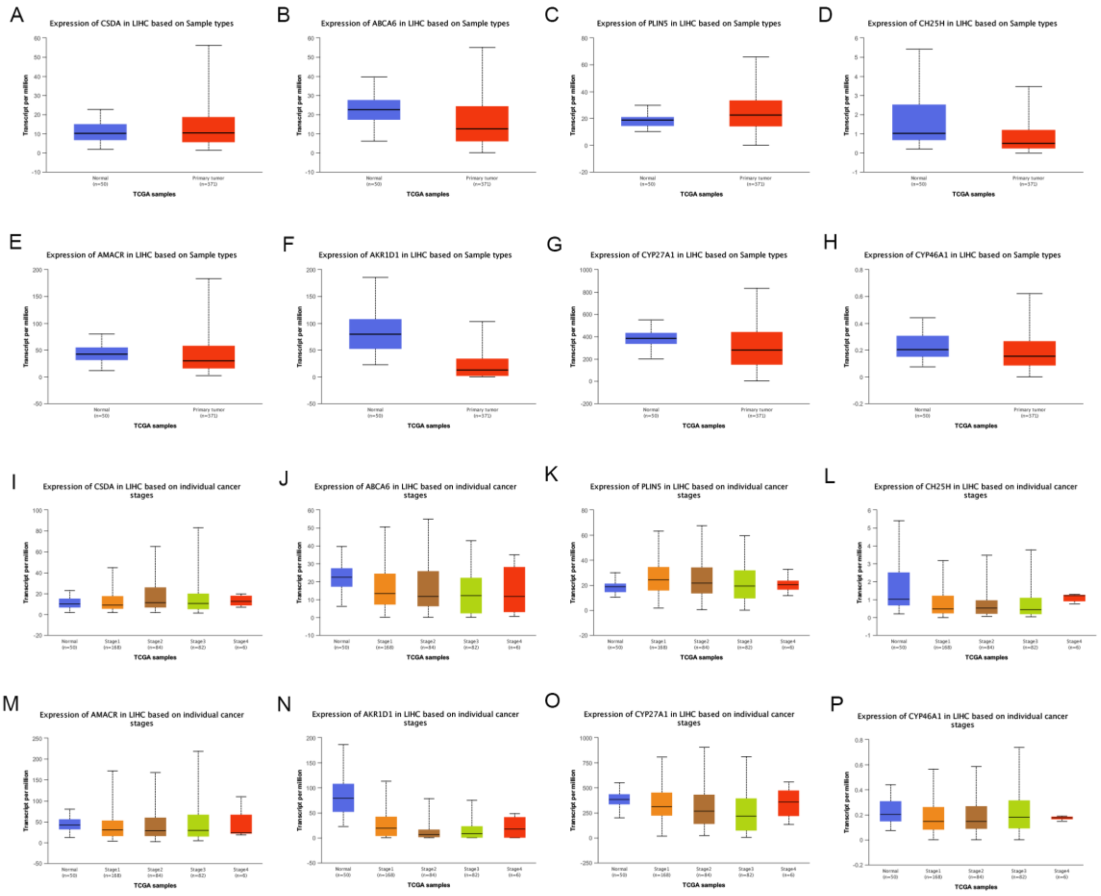

Figure S1 ABCA6, PLIN5, AMACR, AKR1D1 CYP27A1 and CYP46A1 are differently expressed genes among clinical stages in UALCAN.

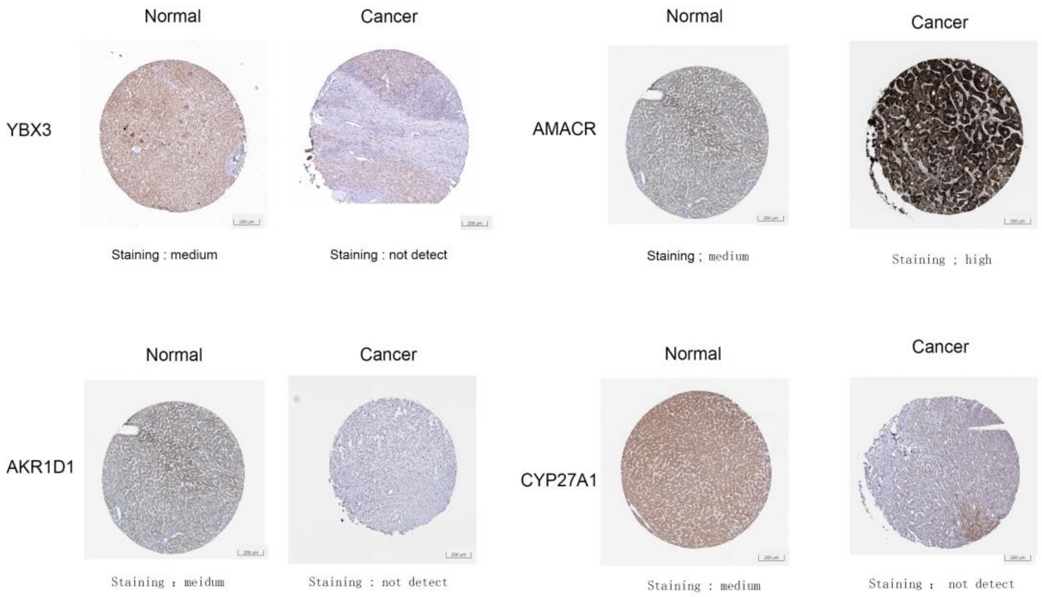

Figure S2 In the Human Protein Atlas, the expression of YBX3, AKR1D1 and CYP27A1 are lower in HCC than normal liver but the AMACR are opposite.

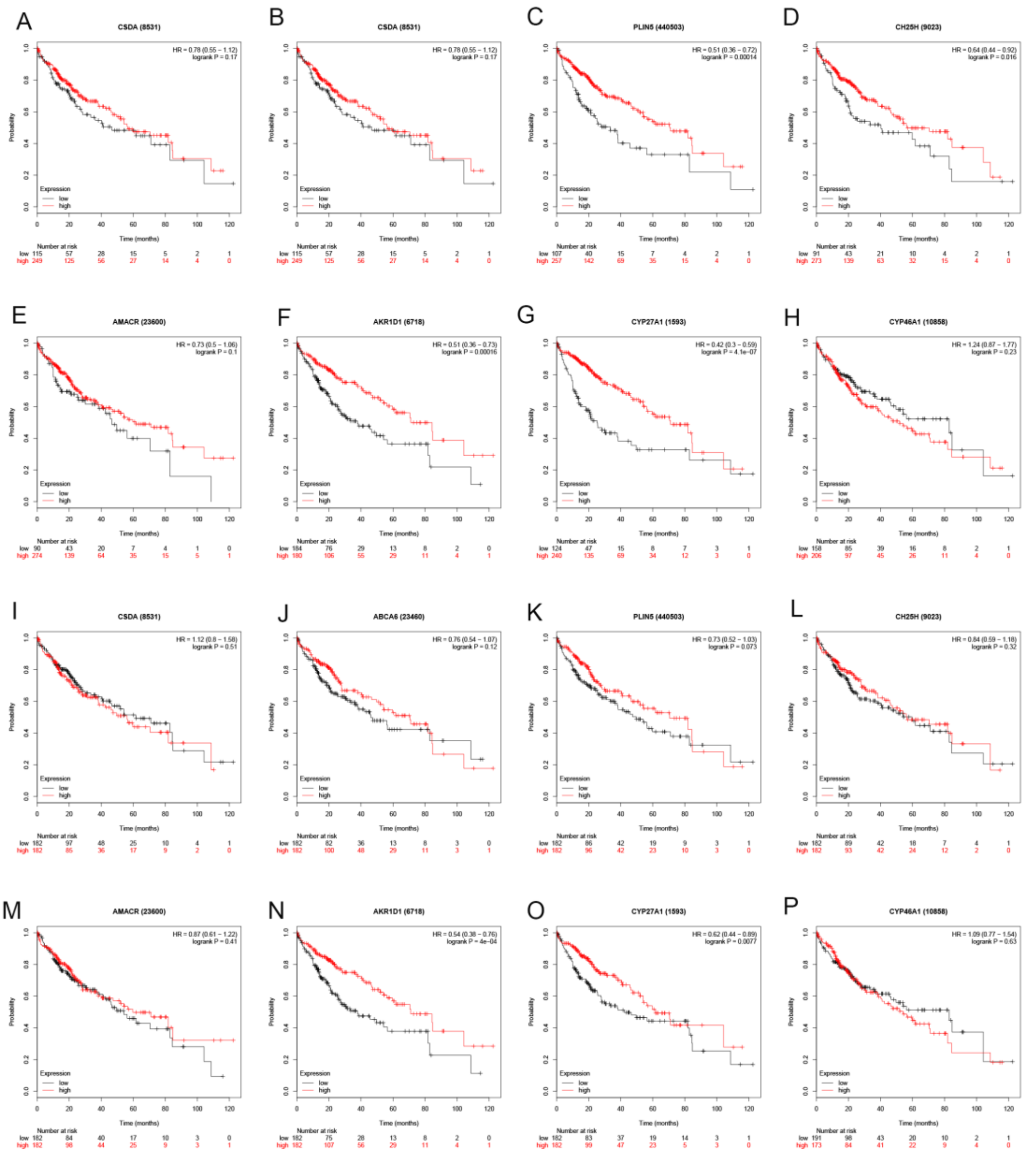

Figure S3 Spliting patients by best cutoff, ABCA6, PLIN5, CH25H, AKR1D1 and CYP27A1 were all related to survival. Spliting patients by median, AKR1D1 and CYP27A1 were related to survival.

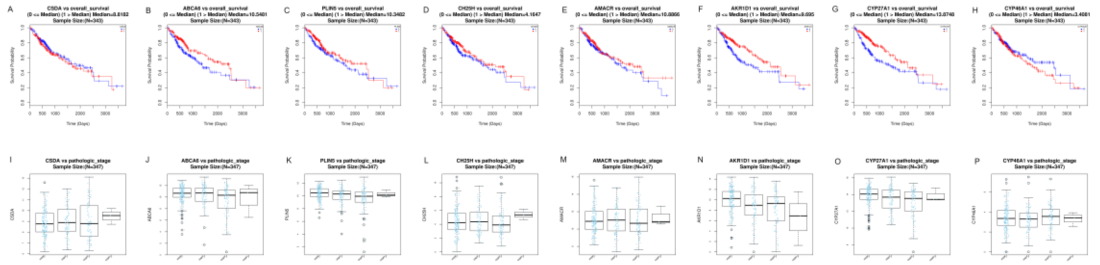

Figure S4 The external validation of YBX3, ABCA6, PLIN5, CH25H, AMACR, AKR1D1, CYP27A1, CYP46A1 at the translational level in Linkedomics. As shown, ABCA6, PLIN5, AKR1D1, CYP27A1 was significantly related with survival and clinical stage.

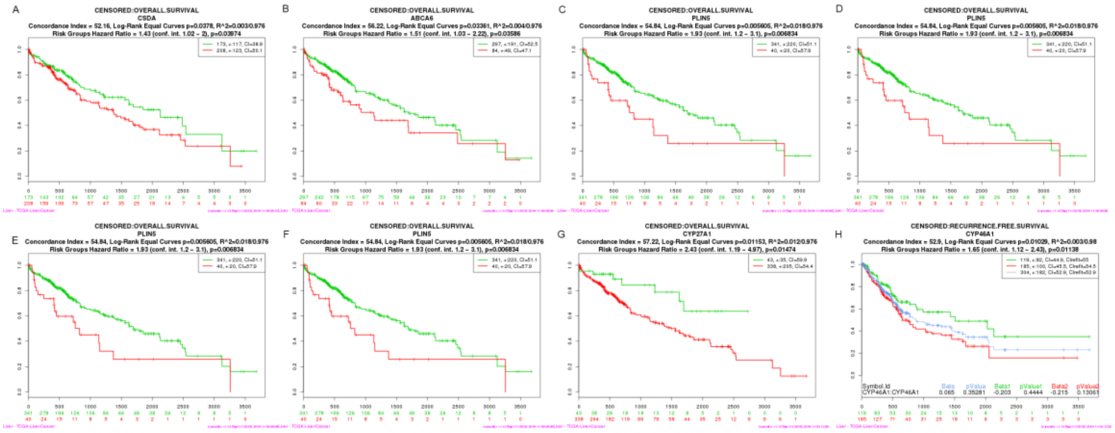

Figure S5 At the tissue level, YBX3, ABCA6, PLIN5, CH25H, AMACR, AKR1D1, CYP27A1, CYP46A1 were all significantly related to survival in SurvExpress.

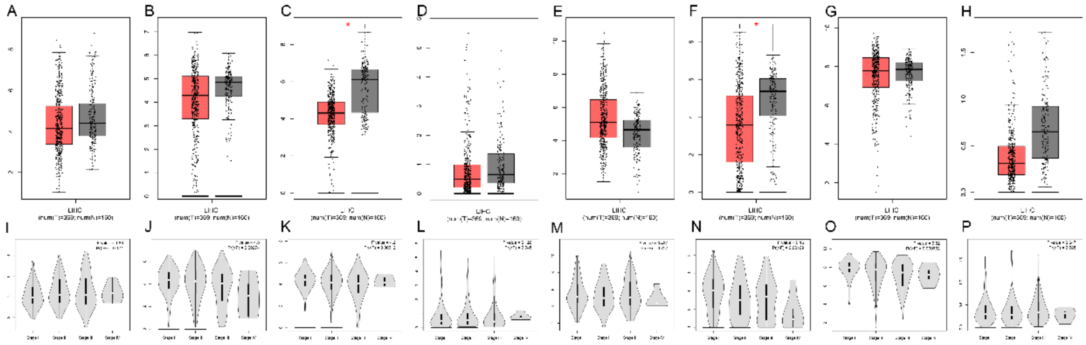

Figure S6 The differential expression of ABCA6 ,PLIN5, AKR1D1, CYP27A1 are related with survival and clinical stages in GEPIA.

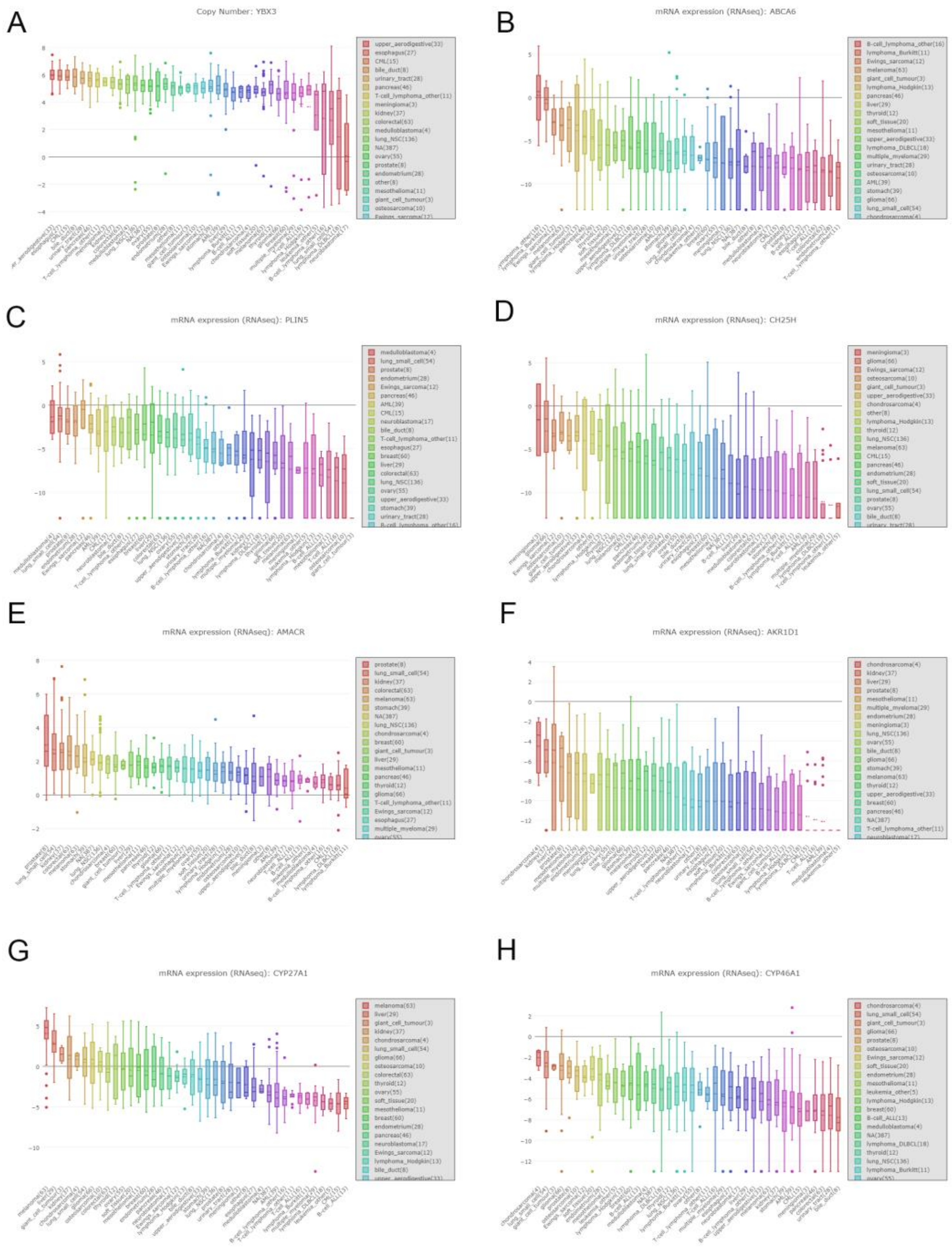

Figure S7 At the cellular level, YBX3 and CH25H were high expressed in liver cancer cell lines, and ABCA6, PLIN5, AMACR, AKR1D1, CYP27A1, CYP46A1 were high expressed in liver cancer cell lines in CCLE.

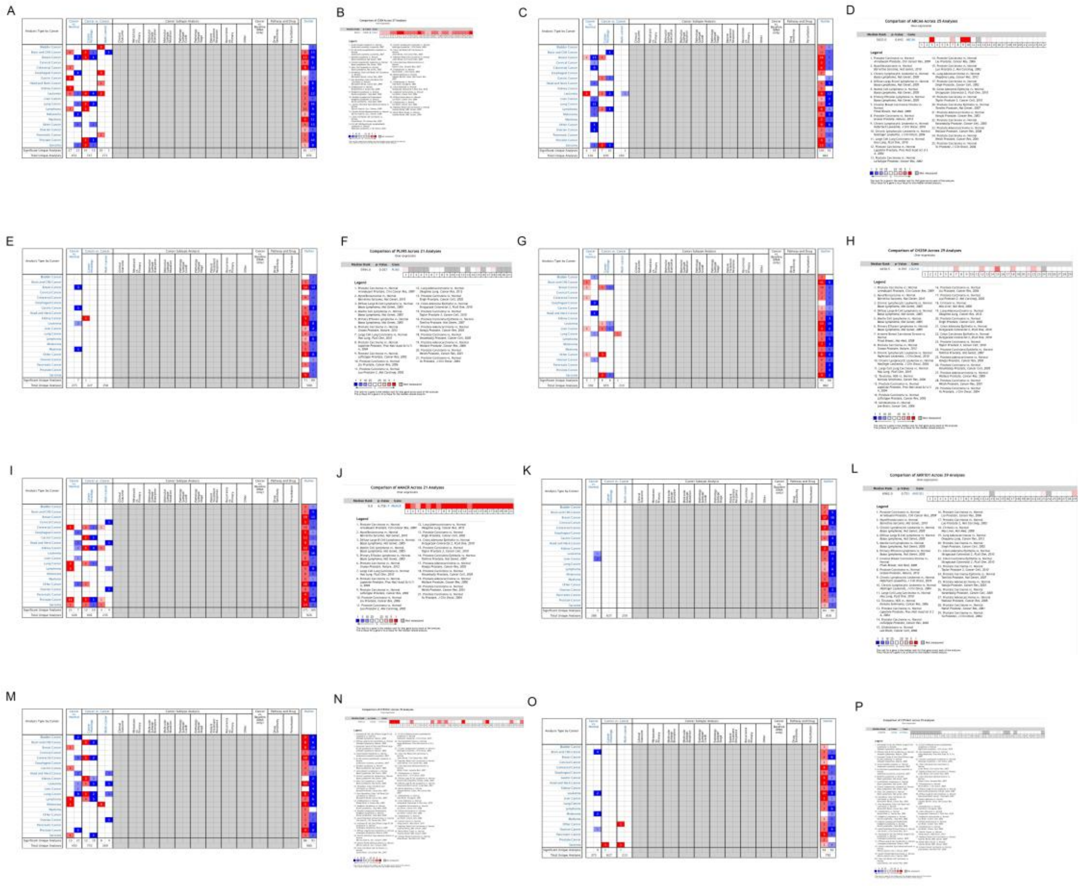

Figure S8 The differential expression of PLIN5, AMACR and YBX3 shows in multiple studies and multiple cancers in oncomine.

Table S1 Baseline information of 377 patients diagnosed with liver hepatocellular carcinoma

Variables	Total Patients (N=377)
**Age, years**	
Mean ± SD	59.45 ± 10.56
Median (Range)	61.00 (16 - 90)
**Gender**	
Female	122 (32.36%)
Male	255 (67.64%)
**Metastasis**	
Yes	41 (10.88%)
No	336 (89.12%)

Abbreviations :SD, Standard deviation.
